# Impact of Immune-Related Adverse Event Frequency, Severity and Corticosteroid Use on Immune Checkpoint Inhibitor Efficacy in Non-Small Cell Lung Cancer

**DOI:** 10.3390/cancers18101538

**Published:** 2026-05-09

**Authors:** José del Corral-Morales, Carlos Ayala-de Miguel, Laura Quintana-Cortés, Santiago González-Santiago, José Fuentes-Pradera, Pablo Ayala-de Miguel

**Affiliations:** 1 Medical Oncology Department, Hospital de Mérida, 06800 Badajoz, Spain; 2 Medical Oncology Deparment, Complejo Hospitalario Universitario de Cáceres, 10004 Cáceres, Spain; carlos.ayala@salud-juntaex.es (C.A.-d.M.); santiago.gonzalez@salud-juntaex.es (S.G.-S.); pablo.ayala@salud-juntaex.es (P.A.-d.M.); 3 Medical Oncology Department, Hospital Don Benito-Villanueva, 06400 Badajoz, Spain; laura.quintana@salud-juntaex.es; 4 Medical Oncology Department, Hospital Universitario Virgen de Valme, 41014 Sevilla, Spain; jose.fuentes.pradera.sspa@juntadeandalucia.es

**Keywords:** carcinoma, non-small-cell lung cancer, immunotherapy, drug-related side effects and adverse reactions, immune-related adverse events

## Abstract

Background/Objectives: Immune checkpoint inhibitors (ICIs) have transformed the treatment landscape of non-small cell lung cancer (NSCLC), introducing a distinct spectrum of immune-related toxicities (irAEs). However, the real-world impact of irAEs and corticosteroid use on treatment outcomes remains uncertain. Methods: We conducted a multicenter, retrospective study including patients with advanced NSCLC treated with ICIs alone or in combination with chemotherapy between April 2017 and December 2023. Clinical records were reviewed to collect data on irAEs and corticosteroid administration. Efficacy and safety outcomes were compared according to irAE occurrence, grade, and corticosteroid use. Categorical variables were analyzed using chi-square tests, while PFS and OS were estimated using the Kaplan–Meier method and compared with the log-rank test. Cox proportional hazards models were used to estimate HRs and 95% CIs. Results: Among 452 patients, 151 (33.4%) experienced irAEs of any grade, and 37 (8.2%) developed grade ≥3 events. The most common irAEs were dermatologic (11.1%), endocrine disorders (9.1%), and arthritis (5.5%). Corticosteroids were administered for irAE management in 60 patients (13.3%). Patients who developed irAEs achieved significantly improved progression-free survival (median PFS: 23.2 vs. 4.2 months; HR = 0.29; *p* < 0.001) and overall survival (median OS: 31.2 vs. 7.6 months; HR = 0.35; *p* < 0.001), including those with grade ≥3 events. The survival benefit associated with irAEs was not compromised by corticosteroid (CS) use for irAE management (median PFS 46.3 in irAE vs. 5.5 months in non-irAE/no use; HR = 0.28; *p* < 0.001). Temporary ICI discontinuation due to irAEs was associated with longer median PFS (39.1 vs. 5.6 months; HR = 0.29; *p* < 0.001), as was permanent ICI discontinuation due to irAEs (median not reached vs. 7.4 months; HR = 0.20; *p* < 0.001). Conclusions: In this retrospective cohort, the occurrence of irAEs—and their management with CS—was associated with enhanced ICI efficacy in metastatic NSCLC, including among patients who developed high-grade toxicities and those who discontinued treatment due to toxicity.

## Simple Summary

Immunotherapy has transformed the treatment of non-small cell lung cancer, but it has also introduced a new spectrum of side effects caused by immune system activation. This study evaluates whether the frequency and severity of these immune-related adverse events are associated with treatment efficacy and whether the use of corticosteroids to manage toxicity may influence clinical benefit. By analyzing data from 452 patients, we also assessed whether discontinuing immunotherapy due to toxicity negatively affects outcomes. Our findings suggest that the occurrence of immune-related adverse events may be linked to improved efficacy, while treatment discontinuation, when required, does not necessarily compromise benefit. These results may help clinicians better understand how to manage immune-related toxicities and make informed decisions, particularly in patients experiencing more severe events.

## 1. Introduction

Immune checkpoint inhibitors (ICIs) have transformed the treatment landscape of non-small cell lung cancer (NSCLC). In metastatic disease, anti-PD-1 and anti-PD-L1 agents—including pembrolizumab, nivolumab, atezolizumab, cemiplimab, and durvalumab—are widely used in the first-line setting, either alone or together with platinum-based chemotherapy, according to PD-L1 expression and other clinical factors [1,2].

The use of ICIs has also introduced a new spectrum of irAEs, which require an appropriate evaluation and patient management [3]. These toxicities are classified using the National Cancer Institute common toxicity criteria for adverse events (NCI CTCAE) [4]. Grade ≥3 toxicities are potentially life-threating and may require permanent discontinuation and prolonged administration of high-dose steroids [5]. However, several studies have reported that patients with NSCLC who develop irAEs during ICI therapy may experience improved survival outcomes [3].

Corticosteroids (CS) are widely used not only for managing irAEs but also for controlling cancer-related symptoms unrelated to immunotherapy. However, their immunosuppressive effect may alter ICI efficacy. Notably, patients receiving ≥10 mg prednisone equivalent were excluded from pivotal clinical trials [6]. Although the impact of CS on ICI efficacy remains controversial, retrospective studies have suggested that doses of ≥10 mg/day prednisone equivalent for non-irAE indications may be associated with poorer outcomes [7,8]. In contrast, real-world data suggest that steroids due to irAEs do not interfere with ICI efficacy, with some controversies regarding the dose, which may lead to reconsider starting with lower dose whenever is feasible [9,10].

In this multicenter retrospective study, we aimed to explore the relationship between irAE and treatment outcomes in a large cohort of patients with advanced NSCLC treated with ICIs. Additionally, we assessed the effect of CS use—both for irAE and non-irAE indications—on progression-free survival (PFS) and overall survival (OS) in this population.

## 2. Materials and Methods

### 2.1. Study Design and Participants

This was a multicenter retrospective study of patients with histologically confirmed locally advanced or metastatic non-small cell lung cancer (NSCLC) treated with immune checkpoint inhibitors (ICIs) targeting PD-1 or PD-L1. Eligible patients had received atezolizumab, nivolumab, pembrolizumab, or cemiplimab, either as monotherapy or combined with platinum-based chemotherapy, as part of routine clinical care. Clinical data were obtained from four Spanish hospitals located in Extremadura and Andalucía. The study period covered patients who started ICI-based treatment between April 2017 and December 2023, with a data cutoff on 10 April 2025.

Patients were excluded if they had received fewer than two ICI administrations or if follow-up information was insufficient to evaluate efficacy outcomes. Immune-related adverse events (irAEs) were identified retrospectively at each center through review of electronic health records, including oncology visits, physician-documented toxicity assessments, treatment interruptions or discontinuations, corticosteroid prescriptions when available, emergency department evaluations, and hospital admissions. Toxicities were graded according to the Common Terminology Criteria for Adverse Events (CTCAE), version 5.0, based on the information documented by the treating physicians. The irAE categories considered in the analysis are listed in the Results section.

Corticosteroid exposure was collected separately according to indication, distinguishing corticosteroids prescribed for irAE management from those used for other medical reasons. Corticosteroids administered exclusively as chemotherapy premedication were not considered. To allow comparison across different agents, doses were converted into dexamethasone-equivalent doses. The cutoff of ≤4 mg versus >4 mg was based on the median corticosteroid dose observed in the study population; this dose corresponds approximately to 25 mg of prednisone, allowing a clinically meaningful interpretation.

Temporary ICI interruption was defined as treatment withholding due to an irAE, followed by ICI rechallenge after toxicity improvement or resolution. Permanent ICI discontinuation was defined as ICI cessation due to recurrent or severe irAEs without subsequent reintroduction. Treatment discontinuations attributable to disease progression, clinical deterioration, or other non-toxicity-related causes were not classified as irAE-related discontinuations.

The study was performed in accordance with the Declaration of Helsinki and was approved by the Ethics Committee of Complejo Hospitalario Universitario de Cáceres (Reference: 018-2024).

### 2.2. Objectives

The main objective was to assess whether the development, severity, and number of irAEs were associated with ICI efficacy in patients with locally advanced or metastatic NSCLC. Efficacy was evaluated using objective response rate (ORR), progression-free survival (PFS), and overall survival (OS). A further objective was to explore whether corticosteroid use for irAE treatment modified the association between irAEs and clinical outcomes.

PFS was calculated from the date of ICI initiation to radiologically documented disease progression or death from any cause, whichever occurred first. OS was defined as the interval between the start of immunotherapy and death from any cause. ORR included complete response (CR) and partial response (PR), whereas disease control rate (DCR) included CR, PR, and stable disease (SD). Tumor burden was estimated as the sum of the longest diameters of up to five target lesions, with no more than two lesions per organ. Radiological response assessments were performed according to local institutional practice, without centralized radiological review.

### 2.3. Statistical Analysis

Patients without disease progression or death at the time of analysis were censored at their last documented follow-up. Categorical variables were described using absolute and relative frequencies. Unless otherwise indicated, continuous variables were categorized according to the median value. Associations between categorical variables were examined using cross-tabulation analyses and the chi-square test.

PFS and OS were estimated using the Kaplan–Meier method, and median survival times were reported. Survival distributions between groups were compared using the log-rank test. Cox proportional hazards regression models were used to estimate hazard ratios (HRs), 95% confidence intervals (CIs), and *p*-values. The proportional hazards assumption was evaluated by visual inspection of log-minus-log survival plots.

Univariable Cox regression analyses were first performed for candidate prognostic factors. Variables showing statistical significance in univariable analysis, together with clinically relevant covariates including histopathological features, treatment-related factors, and laboratory parameters, were entered into multivariable Cox regression models to account for potential confounding.

Analyses were conducted according to irAE occurrence, corticosteroid indication, irAE frequency, and maximum irAE grade. For the assessment of irAE frequency, patients were categorized according to the total number of irAE episodes recorded, irrespective of severity. For severity analyses, patients were classified according to the highest-grade irAE documented during treatment.

Given the time-dependent nature of irAEs, landmark analyses were performed at 12, 24, 36, and 48 weeks after ICI initiation to reduce immortal time bias. This approach was also intended to minimize potential misclassification between early chemotherapy-related toxicities and immune-mediated events in patients receiving chemo-immunotherapy.

All tests were two-sided, and *p*-values <0.05 were considered statistically significant. Statistical analyses were performed using IBM SPSS Statistics, version 27.0.1 (IBM Corp., Armonk, NY, USA).

### 2.4. Review

To contextualize the findings, a narrative review of the literature was conducted using PubMed. The search was limited to English-language articles available up to May 2026 and focused on studies addressing NSCLC, immune-related adverse events, corticosteroid exposure, and ICI efficacy. The search terms included “non-small cell lung cancer”, “immune-related adverse events”, “corticosteroids”, and “efficacy”. References were selected according to their relevance to the study question, and additional publications were retrieved through manual review of reference lists.

## 3. Results

### 3.1. Patient Characteristics

Among patients screened during the study period, 452 with locally advanced or metastatic NSCLC treated with immunotherapy met the eligibility criteria and were included in the final analysis. Baseline demographic and disease characteristics are presented in Table 1. The cohort had a median age of 67 years (range, 40–89), with a predominance of male patients (82.1%) and adenocarcinoma histology (58.4%). After stratification by irAE occurrence, several baseline imbalances were observed. Patients in the non-irAE group more frequently had M1c disease according to the 8th TNM edition (42.5% vs. 31.1%) and bone metastases (29.1% vs. 19.4%). Conversely, PD-L1 expression ≥50% was more frequent among patients who developed irAEs (55.7% vs. 31.6%).

### 3.2. Characterization of irAEs

Among the 452 patients included in the study, 151 (33.4%) developed an irAE of any grade, of whom 35 (7.7% of the total cohort) experienced grades ≥3 irAEs. The most frequently observed irAEs we dermatologic disorders (11.1%), endocrine disorders (9.1%), and pneumonitis (5.5%) as detailed in Table 2. Regarding the number of irAE episodes, 6 patients (1.3% of the total cohort) experienced ≥3 episodes and 35 patients (7.7%) experienced ≥2 episodes. Four treatment-related deaths were reported: one due to severe dermatologic toxicity, two due to hepatotoxicity, and one due to pneumonitis.

The median number of treatment cycles before the onset of the first irAE was 4 (range: 1–115). ICIs were temporarily discontinued in 74 patients (16.3%), and 35 patients (7.7%) required permanent discontinuation due to persistent grade 2 or grade 3–4 irAEs.

Corticosteroids (CS) were used exclusively for the treatment of irAEs in 60 patients (13.3%) during ICI therapy, and for both irAEs and other medical conditions in 15 (3.3%) patients. Of the overall population, 17.0% of patients were receiving CS within 2 months before ICI initiation, and 26.5% within 1 month before ICI initiation. The median CS dose in the irAE group was 4 mg dexamethasone-equivalent (range: 0.8–75 mg) and median treatment duration was 45 days (range: 3–330). Similarly, patients receiving CS for both irAEs and other indications (n = 15) had a median dexamethasone-equivalent dose of 4 mg (range: 1–20 mg) and a median treatment duration of 40 days (range: 19–150). In the group treated for other indications (n = 155), the median CS dose was 4 mg (range: 0.5–24 mg), and the median treatment duration was 30 days (range: 1–360).

### 3.3. Impact of irAEs on Efficacy of ICIs

The DCR and ORR for the entire cohort were 57.7% and 36.9%, respectively, with median PFS and OS of 7.1 and 13 months. DCR and ORR at the first radiologic evaluation were 60.4% and 31.9%, respectively. Patients who developed irAEs (n = 151) had significantly higher DCR (88.1% vs. 42.5%; *p* < 0.001) and ORR (62.3% vs. 24.3%; *p* < 0.001) than those without irAEs (n = 301).

**Figure 1:**
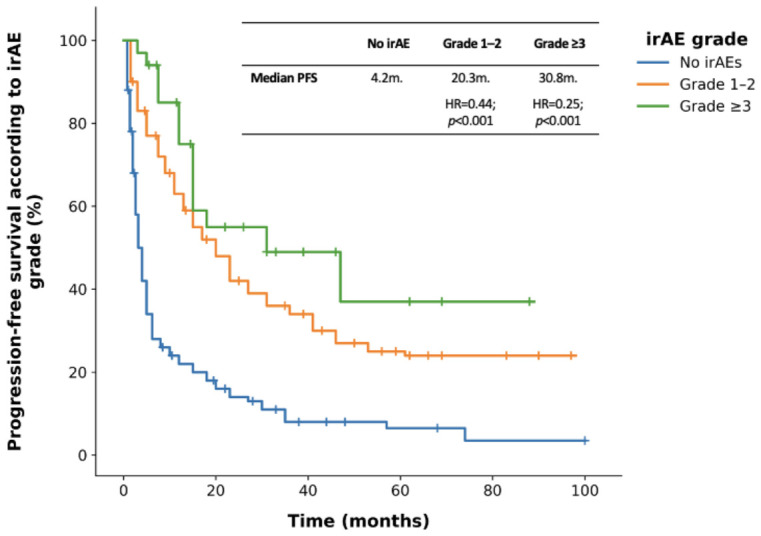
Progression-free survival according to irAE grade. *p*-values were calculated using the log-rank test.

Patients who experienced irAEs showed significantly improved survival outcomes. Median PFS in the irAE group was 23.2 months vs. 4.2 months in the non-irAE group (hazard ratio [HR] = 0.29; CI: 0.23–0.38; *p* < 0.001). Both patients with grade ≥3 and grade 1–2 irAEs had significantly longer PFS than those without irAEs (47.2 vs. 4.2 months, HR = 0.21, 95% CI: 0.12–0.37, *p* < 0.001; and 21.3 vs. 4.2 months, HR = 0.31, 95% CI: 0.24–0.41, *p* < 0.001, respectively) (Figure 1).

**Figure 2:**
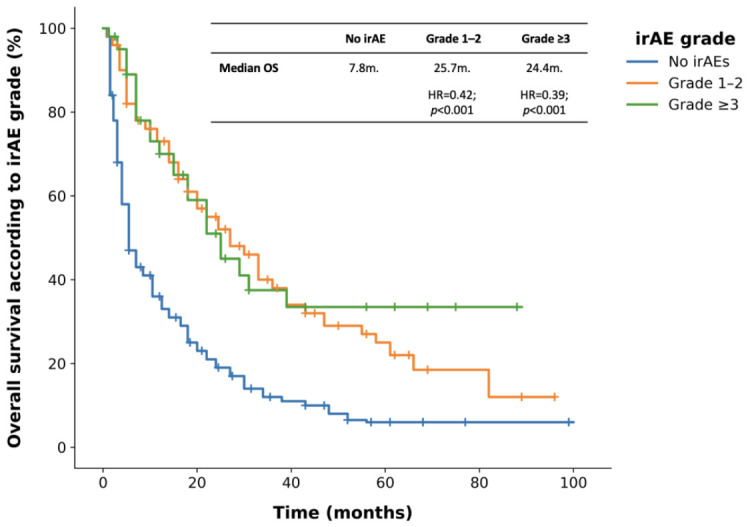
Overall survival according to irAE grade. *p*-values were calculated using the log-rank test.

A statistically significant difference in OS was also observed. Median OS in the irAE group was 31.2 months vs. 7.6 months in the non-irAE group (HR = 0.35; 95%. CI: 0.27–0.44; *p* < 0.001). Patients with grade ≥3 irAEs had significantly longer OS than those without irAEs (24.4 vs. 7.6 months; HR = 0.37; 95% CI: 0.23–0.57; *p* < 0.001), and those with grade 1–2 irAEs similarly showed improved OS (33 vs. 7.6 months in non-irAE; HR = 0.34; 95% CI: 0.26–0.45; *p* < 0.001) (Figure 2).

A 12-week landmark analysis including 323 patients (139 with irAEs) confirmed these findings in favor of the irAE group, demonstrating significantly longer median PFS (27.5 vs. 6.8 months in the non-irAE group; HR = 0.34; 95% CI: 0.26–0.45; *p* < 0.001) and OS (33.6 vs. 14.2 months in the non-irAE group; HR = 0.41; 95% CI: 0.31–0.54; *p* < 0.001).

To mitigate immortal time bias, additional landmark analyses at 24, 36, and 48 weeks were performed. At 24 weeks from ICI initiation, 127 of 225 patients had experienced at least one irAE and demonstrated significantly longer PFS compared with those without irAEs (30.8 vs. 15.4 months; HR = 0.53; 95% CI: 0.38–0.74; *p* < 0.001). At 36 weeks, 105 of 172 patients had developed irAEs and also showed significantly longer PFS compared with those without irAEs (35.5 vs. 22.8 months; HR = 0.66; 95% CI: 0.44–0.99; *p* = 0.043). At 48 weeks, 92 of 147 patients had developed irAEs, with a non-significant trend toward longer PFS (median PFS: 39.4 vs. 28.1 months; HR = 0.69; 95% CI: 0.42–1.11; *p* = 0.123).

Patients with ≥2 irAE episodes (n = 36) had longer PFS than those without irAEs (46.3 vs. 4.2 months; HR = 0.24; 95% CI: 0.16–0.35; *p* < 0.001), and a single irAE episode (n = 112) was also associated with prolonged PFS compared to the no-irAE group (21.1 vs. 4.2 months; HR = 0.38; 95% CI: 0.29–0.51; *p* < 0.001). Moreover, ≥2 irAE episodes conferred longer PFS than a single episode (46.3 vs. 21.1 months; HR = 0.54; 95% CI: 0.30–0.96; *p* = 0.037).

**Figure 3:**
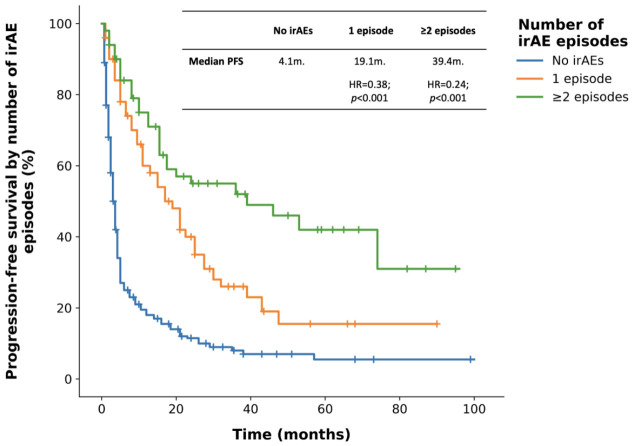
Progression-free survival according to ICI discontinuation due to an immune-related adverse event (irAE). *p*-values were calculated using the log-rank test.

Among patients who temporarily discontinued ICIs (n = 74), a clinical benefit was observed, with median PFS of 39.1 vs. 5.6 months in patients who did not discontinue (HR = 0.29; 95% CI: 0.20–0.42; *p* < 0.001). In particular, patients who permanent discontinued due to an irAE had longer PFS than those who did not discontinue ICIs due to irAEs, including patients still on therapy or those who discontinued for progression or death. Median PFS was not reached (NR) in this group vs. 7.4 months in the control group (HR = 0.20; 95% CI: 0.10–0.41; *p* < 0.001) (Figure 3).

### 3.4. Impact of Corticosteroids on Effectiveness of ICIs

Patients who received CS for irAE management experienced longer PFS (46.3 vs. 5.5 months; HR = 0.28; 95% CI: 0.19–0.40; *p* < 0.001) and OS (32.6 vs. 10 months; HR = 0.38; 95% CI: 0.28–0.54; *p* < 0.001) compared to those who did not.

Stratifying by the occurrence of irAEs, patients treated with CS demonstrated longer median PFS than those with irAEs who did not receive CS (46.3 vs. 19.1 months; HR = 0.56; 95% CI 0.36–0.89; *p* = 0.013). Further efficacy details regarding CS use in this cohort are provided in Table 3.

No significant differences in PFS were observed according to dexamethasone-equivalent dose among patients receiving CS for irAEs (n = 75). Median PFS was NR in patients receiving ≤4 mg dexamethasone-equivalent vs. 15.9 months in those receiving >4 mg DEX-equivalent dose (HR = 0.50; 95% CI 0.24–1.08; *p* = 0.078). Conversely, among patients requiring CS for non-irAE indications (n = 160), dose appeared to influence PFS, with median PFS of 5.6 months in those receiving ≤4 mg vs. 3.4 months in patients receiving >4 mg dexamethasone-equivalent dose (HR = 0.67; 95% CI: 0.46–0.99; *p* = 0.045).

### 3.5. Multivariable Analysis

A Cox regression model confirmed that ECOG 0–1 PDL-1 expression ≥50%, the occurrence of irAEs and the exclusive use of CS for their management were independently associated with longer PFS. Similarly, OS was significantly prolonged in patients with ECOG 0–1, stage III/IV-A, PD-L1 expression >1%, irAEs occurrence and those who received CS exclusively for irAEs. Additional details are provided in Table 4.

## 4. Discussion

Our study, based on a large real-world cohort of patients with advanced NSCLC, demonstrates a statistically significant improvement in ORR, PFS, and OS among those who developed irAEs of any grade. Higher-grade irAEs and multiple episodes were associated with greater clinical benefit. Importantly, CS use for irAE management did not adversely affect ORR, PFS, or OS, thereby adding robust evidence to the current body of literature on the prognostic role of irAEs in ICI-treated patients.

A meta-analysis of randomized clinical trials assessing ICIs—administered either as monotherapy or in combination with placebo, chemotherapy, or other ICIs—in patients with NSCLC reported rates of any-grade and severe irAEs of 37.1% and 18.5%, respectively, along with a discontinuation rate due to severe irAEs of 9.2%. These prospective data closely resemble our findings, which showed irAE rates of 36.9% for any grade and 22.2% for grade ≥3 events, with treatment discontinuation due to persistent grade 2 or grade ≥3 irAEs occurring in 7.7% of patients [11].

Current evidence on the association between irAEs and the efficacy of ICIs in patients with advanced NSCLC remains limited [12]. While some prospective data exist, the majority of findings are derived from retrospective cohort studies [3,13,14,15,16,17,18]. The strongest evidence to date comes from metastatic melanoma, where a secondary analysis of a phase III trial demonstrated a clear association between irAEs and improved clinical outcomes [19]. However, melanoma exhibits unique immunological features, which may limit the generalizability of these results to other solid tumors, including NSCLC.

A non-interventional prospective study including various non-melanoma solid tumors, Schweizer et al. reported significantly longer median PFS (8.8 vs. 4.0 months, *p* = 0.026) and OS (22.8 vs. 11.3 months, *p* = 0.037) in the NSCLC subgroup among patients experiencing irAEs (n = 46) [20]. Similarly, another prospective analysis of 97 patients evaluated the association between irAEs and ICI response, with 51% of patients experiencing irAEs of any grade and 7% experiencing grade ≥3 events. Notably, patients with grade ≥3 irAEs had a higher ORR compared to those with no or only grade 1–2 irAEs (68% vs. 20%, *p* = 0.023) [13]. Another prospective study specifically identified a correlation between ICI-related thyroid dysfunction and longer OS in a cohort of 51 patients with advanced NSCLC enrolled in KEYNOTE-001 [21]. However, these studies primarily included patients receiving ICI monotherapy, with only a small proportion receiving combination therapy [18]. In contrast, our retrospective cohort included 32.9% of patients treated with chemo-immunotherapy, yet showed comparable findings. These results underscore the need for prospective studies specifically evaluating the predictive value of irAEs in the context of combination regimens.

Retrospectively, several meta-analyses with large cohorts reported an association between the occurrence of irAEs and improved response and survival outcomes [22,23,24]. Ma S. et al. meta-analysis, which included 2 prospective studies out of 18, not only evaluated this correlation but also examined how different irAEs may impact ICI efficacy, finding that cutaneous, gastrointestinal, endocrine, and grade ≥3 irAEs were associated with PFS and OS benefit, whereas pneumonitis and hepatotoxicity were not. Moreover, another retrospective study assessed the association between grade 3–4 irAEs and ICI efficacy, showing longer median OS and time to next therapy (TTNT)—as a surrogate of PFS—in patients experiencing grade 3–4 irAEs compared to those without irAEs or with lower-grade events [16], reinforcing the conclusions from our findings. As with prospective studies, retrospective data primarily focused on ICI monotherapy. In one retrospective cohort, where 9 out of 90 patients received chemotherapy, a predictive role of irAEs in ICI efficacy was also observed [6]. Therefore, our findings confirm this association in a larger cohort in which 149 patients received chemoimmunotherapy. Of note, the higher incidence of pneumonitis observed in our cohort may be partly attributable to an underrepresentation of mild-grade irAEs, inherent to the retrospective design.

IrAEs are considered time-dependent events, which is why several retrospective studies have employed landmark analyses to minimize bias [15], particularly in settings where ICIs are administered in combination with chemotherapy. In our study, a landmark analysis was also performed for this purpose, confirming the association between irAEs and improved outcomes. However, meta-analyses [22,23,25] compiling retrospective studies demonstrated a correlation between irAEs and ICI efficacy regardless of whether a landmark analysis was applied. Notably, the aforementioned meta-analyses [22,23,24,25], focused on ICI monotherapy, where chemotherapy-related toxicity could not confound the interpretation of early irAE onset. In contrast, the 12-week cut-off in our study was chosen to ensure that patients had completed the chemotherapy component of combined regimens, thereby minimizing potential misclassification of early toxicities. We acknowledge that this approach may have excluded some early-onset irAEs, which were nonetheless captured in the overall analysis.

Different cutoff points have been used in landmark analyses (e.g., 3, 6, and 12 months) [25,26,27]. In our study, a survival benefit was observed across several cutoff points, thereby reducing the impact of immortal time bias. The lack of statistical significance at the 48-month landmark may be explained by the reduced sample size and the influence of other coexisting favorable prognostic factors.

Several retrospective studies have evaluated the association between the number of irAEs and ICI efficacy and have demonstrated a statistically significant benefit in PFS [15,25,27]. A pooled analysis from the KEYNOTE-001 trial showed that patients with a higher number of irAEs had improved OS (HR = 0.75; 95% CI: 0.58–0.96; *p* = 0.021) and PFS (HR = 0.75; 95% CI: 0.56–0.99; *p* = 0.043) [28]. Our study confirms this association in the largest retrospective cohort to date, observing longer PFS in patients experiencing ≥2 irAE episodes, with significant PFS differences even between patients with ≥2 versus 1 irAE episode.

Regarding CS use, a large real-world study from two institutions reported that doses higher than 10 mg prednisone-equivalent were associated with decreased ORR, PFS, and OS with ICI, even after adjusting for smoking history, ECOG PS, and brain metastases [7]. However, pooled analyses from phase III melanoma trials and a large meta-analysis showed that CS for adverse event management did not negatively impact PFS or OS, whereas their use for symptom control did, highlighting the need for caution [8,27,29]. A retrospective study in patients with stage IV NSCLC reported impaired survival among those treated with ICIs who received high peak doses of CS [30]. Our results are consistent with these findings, showing no impact on PFS or OS when CS were used for irAE management, regardless of dose (≤4 mg or >4 mg dexamethasone-equivalent); however, patients receiving high peak CS doses may be underrepresented in our cohort.

Our study has several limitations. First, the inclusion of patients treated with chemo-immunotherapy raises the possibility that early-onset irAEs may be confounded with chemotherapy-related adverse events. To address this issue, landmark analyses were performed to minimize potential biases, including immortal time bias. Secondly, due to the retrospective nature of the study, some grade 1 irAEs and the exact time to irAE onset may have been underreported or inaccurately captured, potentially leading to an underestimation of their true incidence and limiting more detailed analyses. Morover, irAEs were not analyzed separately by affected organ system, which may have introduced clinical heterogeneity across toxicity types. In addition, baseline differences in ECOG performance status and PD-L1 expression may have influenced survival outcomes; therefore, these variables were considered in multivariable Cox regression analyses to control for potential confounding, although residual confounding cannot be excluded. Finally, the association between ICI discontinuation and longer PFS should be interpreted cautiously, as time-dependent sensitivity analyses could not be performed. Nevertheless, a consistent association between irAEs and survival outcomes was observed across multiple landmark time points. Despite these limitations, our findings provide valuable real-world evidence on the impact of irAEs and CS use on the effectiveness of ICIs.

## 5. Conclusions

In this real-world cohort, the development of irAEs was associated with improved ICI efficacy, even among patients requiring treatment discontinuation or experiencing multiple irAEs. Corticosteroids used for irAE management did not compromise clinical outcomes, regardless of dose. These findings support the prognostic value of irAEs and underscore the need for prospective studies in chemo-immunotherapy settings.

## Figures and Tables

**Table 1:** Patient characteristics. Abbreviations: Squamous cell carc: Squamous cell carcinoma. ICI: Immune-checkpoints inhibitors. ECOG PS: Eastern Cooperative Oncology Group Performance Status.

	Overall, (%) (*n* = 452)	irAEs (%) (*n* = 151)	No irAEs (%) (*n* = 301)	*p*-Value
**Age, y**				0.384
≥65	269 (59.5%)	95 (56.9%)	174 (61.1%)	
<65	183 (40.5%)	72 (43.1%)	111 (38.9%)	
**Sex**				0.814
Male	371 (82.1%)	138 (82.6%)	233 (81.8%)	
Female	81 (17.9%)	29 (17.4%)	52 (18.2%)	
**Smoking status**				0.008
Current smoker	202 (44.7%)	91 (54.5%)	113 (39.6%)	
Never-smoker	40 (8.8%)	11 (6.6%)	29 (10.2%)	
Former smoker	210 (46.5%)	65 (38.9%)	143 (50.2%)	
**Histology**				0.18
Adenocarcinoma	264 (58.4%)	101 (60.5%)	163 (57.2%)	
Squamous cell carc.	152 (33.6%)	50 (29.9%)	102 (35.8%)	
NOS	33 (7.3%)	14 (8.4%)	19 (6.7%)	
Other	3 (0.9%)	2 (1.2%)	1 (0.4%)	
**Stage**				0.197
Recurrent IIIA	17 (3.8%)	6 (3.6%)	11 (3.9%)	
Recurrent IIIB	17 (3.8%)	8 (4.8%)	9 (3.2%)	
Recurrent IIIC	2 (0.4%)	0 (0.0%)	2 (0.7%)	
M1a	158 (34.9%)	64 (38.3%)	94 (33.0%)	
M1b	85 (18.8%)	37 (22.2%)	48 (16.8%)	
M1c	173 (38.3%)	52 (31.1%)	121 (42.5%)	
**M1 location**				
Brain	73 (16.2%)	25 (15.0%)	48 (16.8%)	0.602
Liver	59 (10.1%)	20 (12.0%)	39 (13.7%)	0.603
Bone	115 (25.4%)	33 (19.8%)	82 (28.8%)	0.034
Adrenal	73 (16.2%)	32 (19.2%)	41 (14.4%)	0.183
**PDL1**				<0.001
≥50%	183 (40.4%)	93 (55.7%)	90 (31.6%)	
1–49%	101 (22.4%)	33 (19.8%)	68 (23.9%)	
<1%	101 (22.4%)	23 (13.8%)	78 (27.4%)	
Unknown	67 (14.8%)	18 (10.8%)	49 (17.2%)	
**Treatment line**				0.128
First line	263 (58.4%)	109 (65.3%)	154 (54.0%)	
Second line	153 (33.8%)	50 (29.9%)	103 (36.1%)	
Third and beyond	36 (7.7%)	8 (4.8%)	28 (9.8%)	
**Treatment regimen**				0.008
Chemotherapy-ICI	149 (32.9%)	51 (30.5%)	98 (34.4%)	
Pembrolizumab	134 (29.9%)	65 (38.9%)	69 (24.2%)	
Atezolizumab	116 (25.7%)	33 (19.8%)	83 (29.1%)	
Nivolumab	53 (11.5%)	18 (10.8%)	35 (12.3%)	
**ECOG PS**				0.003
0–1	398 (88.1%)	157 (94%)	241 (84.6%)	
≥2	54 (11.9%)	10 (6.0%)	44 (15.4%)	
**Subsequent therapy**				0.266
0 subsequent line	276 (61.7%)	106 (63.5%)	170 (59.6%)	
1 subsequent line	136 (29.6%)	43 (25.7%)	93 (32.6%)	
≥2 subsequent lines	39 (8.6%)	17 (10.2%)	22 (7.7%)	
**Driver mutations**				0.044
ALK translocation	2 (0.4%)	2 (1.2%)	0 (0.0%)	
EGFR	18 (4%)	2 (1.2%)	16 (5.6%)	
KRAS	52 (10%)	15 (9.0%)	37 (13.0%)	
BRAF	8 (2%)	2 (1.2%)	6 (2.1%)	
MET	3 (0.4%)	1 (0.6%)	2 (0.7%)	

**Table 2:** Immune-related adverse events. Abbreviations: GI: Gastrointestinal.

	Total, n = 452 (%)	Grade 1–2	Grade ≥3
**Affected organ**			
Skin	50 (11.1%)	38 (8.4%)	12 (2.7%)
Endocrine	41 (9.1%)	39 (8.6%)	2 (0.4%)
Arthritis	25 (5.5%)	21 (4.6%)	4 (0.9%)
Pneumonitis	25 (5.5%)	17 (3.8%)	8 (1.8%)
GI	23 (5.1%)	21 (4.6%)	2 (0.4%)
Hepatitis	18 (4.0%)	10 (2.2%)	8 (1.8%)
Nephritis	8 (1.8%)	7 (1.5%)	1 (0.2%)
Mucositis	6 (1.3%)	6 (1.3%)	0 (0.0%)
Neurologic	5 (1.1%)	4 (0.9%)	1 (0.2%)

**Table 3:** Efficacy details according to CS indication. Abbreviations: irAEs: Immune-checkpoint inhibitors; DCR: Disease control rate; ORR: Objective Response Rate; PFS: Progression-free survival; HR: Hazard Ratio. *p*-values were calculated using the chi-square test for categorical variables, the log-rank test for Kaplan–Meier comparisons, and univariable Cox regression for HR estimates.

	irAEs	irAEs and Others	Others	*p*-Value
DCR	90%	93.3%	49.4%	<0.001
ORR	65%	60%	31.3%	<0.001
PFS, months	46.3	21.3	4.5	–
HR for PFS vs. others (95% CI)	0.26 (0.17–0.41)	0.39 (0.18–0.83)	Reference	<0.001 and 0.014

**Table 4:** Multivariable analysis. Abbreviations: PFS: Progression-free survival. OS: Overall survival. HR: Hazard Ratio. Squamous cell carc: Squamous cell carcinoma. ECOG: Estern Cooperative Oncology Group. NLR: Neutrophil–Lymphocyte ratio. irAEs: Immune-related adverse events. CS: Corticosteroids. Note: bold indicates statistical significance at *p* < 0.05. Multivariable Cox regression models included variables that were statistically significant in univariable analyses, together with clinically relevant variables selected as potential confounders, as described in the Methods section.

	PFS		OS	
	**HR**	***p*-Value**	**HR**	***p*-Value**
**Sex**				
Male				
Female	1.12 (0.71–1.75)	0.639	1.04 (0.67–1.63)	0.857
**Age**				
<65				
≥65	0.94 (0.64–1.39)	0.753	1.26 (0.86–1.85)	0.242
**Histology**				
Squamous cell carc.				
Adenocarcinoma	1.11 (0.70–1.76)	0.658	1.15 (0.75–1.78)	0.522
**ECOG**				
0–1				
≥2	2.29 (1.38–3.80)	**0.001**	3.05 (1.86–5.00)	**<0.001**
**NLR**				
<5				
≥5	0.88 (0.60–1.31)	0.533	1.04 (0.71–1.52)	0.830
**Stage**				
III/IVA				
IVB	1.46 (0.95–2.25)	0.083	1.62 (1.08–2.42)	**0.019**
**Brain metastases**				
No				
Yes	0.88 (0.55–1.40)	0.594	0.81 (0.52–1.26)	0.343
**PD-L1 (%)**				
<1%				
1–49%	0.67 (0.40–1.11)	0.123	0.55 (0.34–0.90)	**0.017**
≥50%	0.47 (0.29–0.76)	**0.003**	0.48 (0.30–0.76)	**0.002**
**Treatment line**				
First line				
Second line	0.75 (0.47–1.20)	0.229	0.83 (0.53–1.31)	0.421
Third and beyond	1.13 (0.55–2.35)	0.736	0.93 (0.45–1.93)	0.840
**irAEs**				
No irAEs				
irAEs	0.40 (0.23–0.71)	**0.002**	0.44 (0.26–0.77)	**0.004**
**CS indication**				
Non-irAE				
irAE and non-irAE	0.55 (0.18–1.69)	0.299	0.77 (0.30–2.01)	0.602
irAE	0.49 (0.25–0.97)	**0.041**	0.52 (0.27–0.99)	**0.045**

## Data Availability

The original contributions presented in this study are included in the article. Further inquiries can be directed to the corresponding author.

## Abbreviations

The following abbreviations are used in this manuscript:

ICIs	Immune checkpoint inhibitors
NSCLC	Non-small cell lung cancer
IRAEs	Immune-related adverse events
NCI CTCAE	National cancer institute common toxicity criteria for adverse events
CS	Corticosteroids
ORR	Objective response rate
PFS	Progression-free survival
OS	Overall survival
CR	Complete responses
PR	Partial responses
DCR	Disease control rate
SD	Stable disease
HR	Hazard ratios
CIs	Confidence intervals
ICI	Immune-checkpoints inhibitors
ECOG PS	Eastern cooperative oncology group performance status
GI	Gastrointestinal
NR	Not reached
NLR	Neutrophil–lymphocyte ratio
TTNT	Time to next therapy
